# O083: Prohibit (preventing hospital-acquired infections by intervention and training): preliminary results of a European multi-center study on the effectiveness of a hand hygiene campaign and a central venous catheter bundle

**DOI:** 10.1186/2047-2994-2-S1-O83

**Published:** 2013-06-20

**Authors:** T van der Kooi, M Wolkewitz, B van Benthem, S de Greeff, H Grundmann, W Zingg

**Affiliations:** 1 RIVM, Bilthoven , The Netherlands; 2 Institute of Medical Biometry and Medical Informatics, Freiburg, Germany; 3 University hospitals of Geneva, Geneva, Switzerland

## Introduction

Differences in the incidence of hospital-acquired infections among European hospitals are due to a range of factors such as case mix, variable infection control policies and practices, and culture. The PROHIBIT study aims at inventorying and analysing national and local infection prevention policies and practices in Europe. The aim of this study is to test two interventions of proven efficacy, a hand hygiene (HH) improvement campaign and an extensive central venous catheter (CVC) bundle, in reducing CVC-related bloodstream infection (CRBSI).

## Methods

Fourteen hospitals from 11 countries take part with ≥ one intensive care units (ICU) since January 2011. Primary outcome is CRBSI, secondary outcomes compliance with HH and the CVC bundle. After a baseline of 6 months, every 3 months 3 hospitals were randomized to implement one or both interventions following a stepped wedge design. The last 2 started in July 2012 and the study ends in June 2013. The HH strategy followed the World Health Organization guidelines while the CRBSI prevention strategy is based on a successfull programme of the University of Geneva hospitals.

## Results

The mean (95% confidence interval) baseline CRBSI rate was 2.5/1000 CVC days (2.2-2.8) with an interhospital range of 0.0-10.0. The incidence of most CVC bundle hospitals was ≤1.0 at baseline already. The mean rate upon the intervention was 0.9 (0.8-1.2; range: 0.0-5.1). The mean baseline CVC bundle compliance of 4.3% increased to 40.6% for all hospitals. The mean baseline HH compliance of 48.6% (range: 16.9-67.1), improved to 60.0% for all hospitals (range: 36.1-85.3). Multivariate Cox regression, stratified per hospital, revealed that the HH intervention alone and both interventions combined were associated with reduced CRBSI incidence densities (HR 0.4 and 0.5 respectively).

## Conclusion

Our findings confirm variation of CRBSI incidence densities among European ICUs. The interventions appear effective particularly in hospitals with higher baseline rates. This needs to be confirmed in the final analysis including competing risks and sensitivity analyses.

## Disclosure of interest

None declared.

